# Imaging of singlet oxygen feedback delayed fluorescence and lysosome permeabilization in tumor *in vivo* during photodynamic therapy with aluminum phthalocyanine

**DOI:** 10.1117/1.JBO.25.6.063806

**Published:** 2020-01-09

**Authors:** Marek Scholz, Jason R. Gunn, Geoffrey P. Luke, Brian W. Pogue

**Affiliations:** Dartmouth College, Thayer School of Engineering, Center for Imaging Medicine, Hanover, New Hampshire, United States

**Keywords:** delayed fluorescence, singlet oxygen, photodynamic therapy

## Abstract

**Significance:** Singlet oxygen is a key cytotoxic agent in photodynamic therapy (PDT). As such, its imaging is highly desirable, but existing direct imaging methods are still limited by the exceptionally low yield of the luminescence signal. Singlet oxygen feedback delayed fluorescence (SOFDF) of the photosensitizer is a higher yield alternative for indirect measurement of this signal.

**Aim:** The aim was to explore feasibility of SOFDF imaging *in vivo* in tumor-bearing mice during PDT and investigate how SOFDF images can be transformed into images of singlet oxygen. In addition, we study whether lysosome permeabilization can be visualized through fluorescence lifetime.

**Approach:** Mice were intravenously injected with 2.5 mg/kg of photosensitizer aluminum(III) phthalocyanine tetrasulfonate (${\mathrm{AlPcS}}_{4}$) 20 h prior to experiments, having subcutaneous BxPC3 pancreas tumors. Time-resolved delayed fluorescence and prompt fluorescence (PF) were imaged using an intensified time-gated camera with 10-Hz pulsed laser excitation at 690 nm.

**Results:** Delayed emission from ${\mathrm{AlPcS}}_{4}$ was detected with lifetimes 7 to 11  μs, which was attributed to SOFDF and shown to be oxygen-dependent. Singlet oxygen images were approximated by the ratio of SOFDF/PF at each pixel. SOFDF images of a good quality could be captured within several seconds with a radiant exposure of $∼20\;\mathrm{mJ}/{\mathrm{cm}}^{2}$. In addition, lifetime images of ${\mathrm{AlPcS}}_{4}$ PF in ns-time domain enabled us to visualize the event of lysosome permeabilization, as the lifetime increased from ∼4.7 to 5.2 ns.

**Conclusions:** Imaging of SOFDF *in vivo* in mouse tumor during PDT with ${\mathrm{AlPcS}}_{4}$ is feasible, and it is a promising method for singlet molecular oxygen monitoring. Moreover, the time-gated approach also enables visualization of the lysosome permeabilization that alters the PF lifetime.

## 1 Introduction

Singlet molecular oxygen (O21) is a highly reactive oxygen species produced typically by energy transfer in interaction of ground-state oxygen (O23) with an excited triplet state of a photosensitizer molecule. It is involved in aging and oxidative degradation in general, but it is also a key cytotoxic cell-killing agent in photodynamic therapy (PDT) of cancer and other diseases.^1^^–^^4^ The amount of O21 produced during PDT is believed to be one of the most important factors that predicts success or failure of the treatment. For these reasons, tremendous effort has been dedicated in past several decades to the development of techniques for its detection and imaging. However, only partial success has been achieved, and real-time imaging at conditions relevant to PDT has been elusive so far.^4^ On one side, there is a direct detection of a weak near-infrared phosphorescence of O21. While this is the most unambiguous method, it suffers from very low quantum yields that are in the order of $10^{-7}$ *in vivo*,^5^ and the emission is in the infrared, which makes the detection even more challenging due to limited quantum yield of photocathode detectors and thermal noise in this wavelength range. Although a significant progress has been achieved in point detection,^6^^–^^9^ *in vivo* imaging of O21 phosphorescence still poses a great challenge. Microscopic and macroscopic images in cell cultures and model systems have been reported but usually high light intensities, photosensitizer concentrations, and long exposure times were needed.^10^^–^^13^ In comparison, fluorescence probes that work as chemical traps of O21 were developed, typically reporting on O21 by increase in the fluorescent quantum yield, e.g., Singlet Oxygen Sensor Green and related compounds.^14^^–^^19^ However, these probes were found to suffer from a variety of problems, including cell impermeability, poor specificity, colocalization issues, and incompatibility with translation to human use. Attempts on *in vivo* imaging of O21 with fluorescence probes have been scarce.^14^

**Fig. 1:**
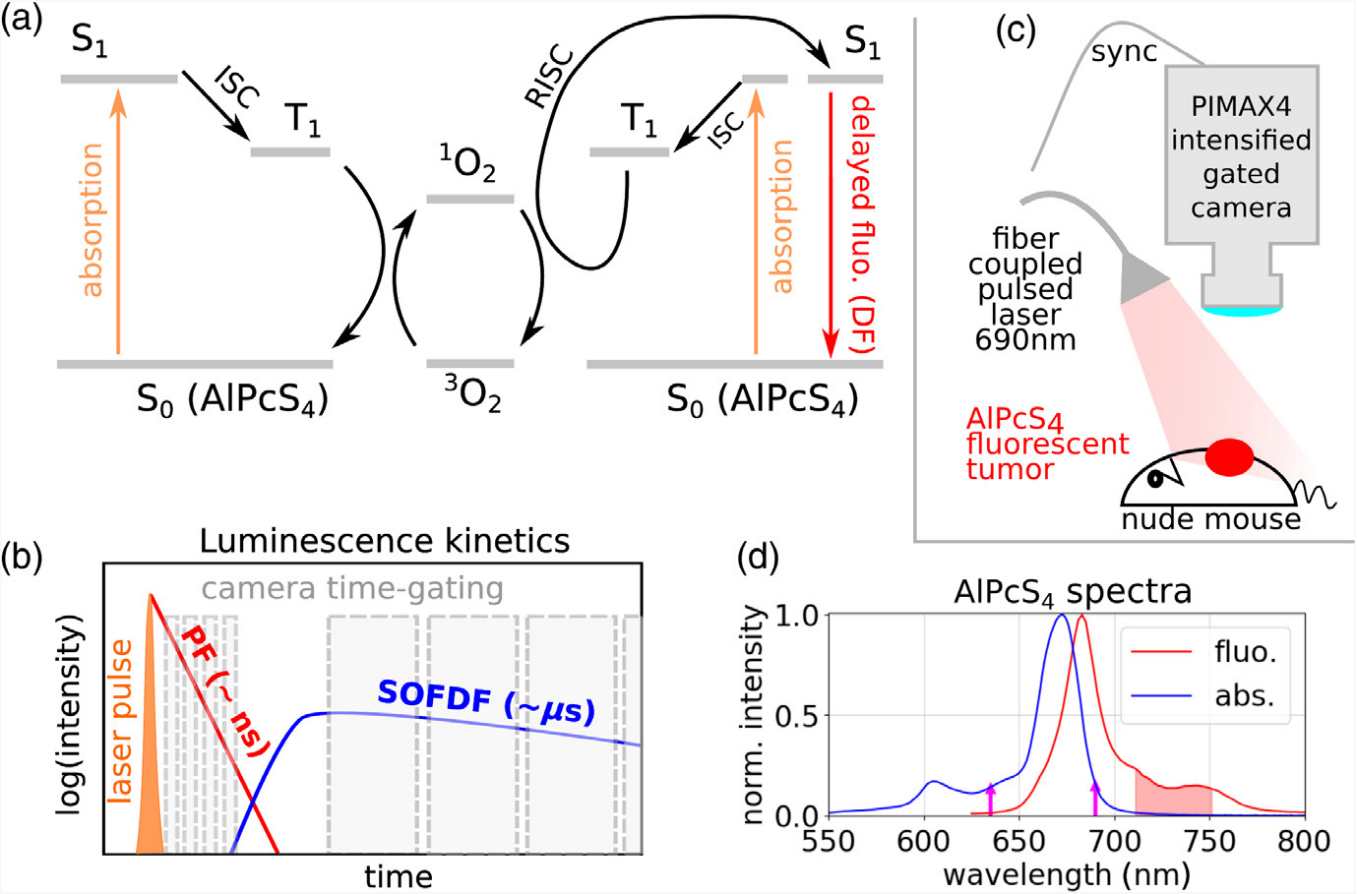
(a) Jablonski diagram of the SOFDF. Two $S_{1}$ states of the photosensitizer absorb a photon and undergo intersystem crossing to the $T_{1}$ state. The $T_{1}$ state on the left is deactivated while interacting with O23 and gives rise to O21. This then diffuses and collides with another $T_{1}$ state and provides energy for RISC, thus repopulating the $S_{1}$ state of the photosensitizer, which can then emit a photon. (b) The sample is excited by a short laser pulse. PF decays in a nanosecond time-scale, whereas DF decays in microsecond time-scale. The emission is detected by a time-gated camera with increasing delay times. Time gate of 3 ns was used for PF lifetime images whereas gate width of several microseconds was used for DF lifetime imaging. (c) Mouse injected with ${\mathrm{AlPcS}}_{4}$ was irradiated with a pulsed laser and signal was collected with an intensified time-gated camera. (d) Absorption and fluorescence spectrum of ${\mathrm{AlPc}}_{4}$ in PBS. The magenta arrows indicate laser wavelengths of 635 and 690 nm with absorptions at 14% and 16% of the maximum. The red area approximates the spectral region collected by the camera.

Recently, we proposed a completely different approach to monitoring singlet oxygen.^20^^–^^22^ It is based on the mechanism of singlet oxygen feedback delayed fluorescence (SOFDF),^23^^–^^25^ which is intrinsic to a number of different photosensitizers (protoporphyrin IX, ionic porphyrins, porphycenes, eosin, Rose Bengal, and others),^20^^,^^26^^–^^28^ and hence no additional probe molecule is required. The triplet state of a photosensitizer ($T_{1}$) can interact with the previously generated O21, which leads to a reverse intersystem crossing (RISC) of the photosensitizer molecule and repopulation of the $S_{1}$ state that can subsequently emit a photon. SOFDF scheme is depicted in Fig. 1(a) and can be summarized as

T1+O23→S0+O21,

O21+T1→O23+S1.

In SOFDF, singlet oxygen thus acts as an intermediate carrier that transfers excitation energy from one triplet to another and provides energy for repopulation of the $S_{1}$ state through RISC. SOFDF intensity is second-order in the concentration of triplet states, because two triplets in total are needed to generate one photon. The rate of SOFDF emission is proportional to the product of triplet concentration and O21 concentration:

ISOFDF(t)∝[T1](t)×[O21](t). (1)

When the triplet decay can be described by one exponential with lifetime $\tau_{T}$, the O21 kinetics is a rise–decay biexponential function with lifetimes $\tau_{T}$ and $\tau_{\Delta }$, where $\tau_{\Delta }$ is the O21 lifetime. *In vivo* typically $\tau_{T}\gg \tau_{\Delta }$, which allows us to express the SOFDF emission rate as^20^^,^^21^

$$I_{\mathrm{SOFDF}}(t)=A[e^{-t/\tau_{T}}\times (e^{-t/\tau_{T}}-e^{-t/\tau_{\Delta }})]\approx A[e^{-t/(\tau_{T}/2)}-e^{-t/\tau_{\Delta }}].$$ (2)

The SOFDF kinetics is then also a biexponential rise–decay function with decay time approximately half of the triplet lifetime and rise-time approximately equal to the O21 lifetime. However, given that intracellular lifetimes of O21 are likely below 1  μs,^29^ it can be challenging to resolve the rise of SOFDF kinetics, especially if background signals are present.^21^ Triplet lifetimes and hence SOFDF decay times are often dictated by the concentrations of O23 that acts as an efficient quencher.

Apart from SOFDF, other mechanisms of delayed fluorescence (DF) have been described,^25^^,^^30^ depending on how the energy for RISC is provided, namely (1) thermally activated DF: $T_{1}\to S_{1}$, and (2) triplet-triplet annihilation DF: $T_{1}+T_{1}\to S_{1}+S_{0}$. There are two main features that distinguish SOFDF from the latter two types of DF: SOFDF kinetics has a biexponential rise–decay shape instead of an exponential decay, and SOFDF disappears in the absence of oxygen, whereas the other types are enhanced. In general, DF and prompt fluorescence (PF) usually have very similar emission spectra. However, DF is a process that happens typically in microsecond time ranges, similar to phosphorescence, whereas PF happens in nanoseconds. DF emission is usually several orders of magnitude weaker than the PF emission.^20^

Aluminum(III) phthalocyanine tetrasulfonate (${\mathrm{AlPcS}}_{4}$) is one of the more potent photosensitizers that emits relatively bright SOFDF.^20^^,^^22^ It is a water-soluble second-generation photosensitizer for PDT of cancer, and it has been commercialized under the name Photosens for treatment of certain types of tumors.^31^^–^^36^ AlPcS_4_ is taken up by endocytosis and localizes in lysosomes.^37^ During irradiation, the generated O21 destabilizes lysosomes, eventually causing lysosome permeabilization and release of its contents into cytosol, including ${\mathrm{AlPcS}}_{4}$.^38^ This event can potentially trigger mechanisms of cell death.^39^ Sulfonated aluminum phthalocyanines have been also investigated in relation to photochemical internalization,^40^ a therapeutic method proposed for a targeted release of chemotherapeutic drugs locally in the irradiated area, which makes use of a photosensitizer-induced lysosome permeabilization.

${\mathrm{AlPcS}}_{4}$ is a brightly fluorescent molecule with peak absorption at ∼673  nm, and fluorescence emission with main peak at ∼680  nm and a side peak at ∼750  nm [Fig. 1(d)].^41^ The quantum yield of PF is around 0.4 in water,^42^^,^^43^ but a part of the excited states undergoes intersystem crossing to the triplet state and then generates O21 by energy transfer with quantum yield of ∼0.3 in solutions.^44^^,^^45^

A detailed investigation of SOFDF *in vitro* in adherent monolayer of fibroblast cells was performed in our recent paper.^22^ It has been shown that ${\mathrm{AlPcS}}_{4}$ emits bright SOFDF while it is localized in lysosomes, and thus reports on the generated O21. After the lysosome permeabilization and release of ${\mathrm{AlPcS}}_{4}$, the SOFDF emission intensity was observed to drop rapidly, whereas the PF intensity increased. These changes in DF and PF were proposed as indicators of lysosomal permeabilization during PDT or photochemical internalization. Here we bring these ideas further and investigate whether DF can provide valuable information also in tumors *in vivo*.

The aim of this work was to explore the feasibility of a time-gated SOFDF imaging *in vivo* in tumor-bearing mice that were intravenously injected with ${\mathrm{AlPcS}}_{4}$. Comparison with the previous results obtained *in vitro* is presented, and benefits and limitations of the time-gated fluorescence imaging for monitoring of singlet oxygen and lysosome permeabilization during PDT are discussed.

## 2 Materials and Methods

Aluminum (III) phthalocyanine chloride tetrasulfonic acid (AlPcS-834, >95%, Frontier Scientific) was used as received without further purification. Tissue phantoms consisted of 50  μM ${\mathrm{AlPcS}}_{4}$, 1% intralipid (Fresenius Kabi), and 1% bovine whole blood (Lampire) in phosphate-buffered saline solution (PBS).

Athymic nude mice were implanted with $1\times 10^{6}$ BxPC3, a human pancreatic tumor cell line, mixed 1:1 with Matrigel in 200  μl. The tumor grew on the rear flank of the mouse subcutaneously. The desired size of tumors (∼150  mm) was reached after 2 to 3 weeks. Then the mice were intravenously injected with ${\mathrm{AlPcS}}_{4}$ dissolved in PBS to obtain a dose of 2.5 mg/kg.^33^ Imaging was performed 20 h after the injection. All *in-vivo* procedures in this work followed a protocol approved by the Institutional Animal Care and Use Committee.

To detect both time-resolved DF and PF, an intensified gated camera PIMAX4 (Princeton Instruments) was used together with a pulsed laser [Figs. 1(b) and 1(c)]. The camera enabled gate widths ranging from 3 ns up to seconds. An Nd:YAG second-harmonic pumped optical parametric oscillator laser (Opotek Phocus Mobile HE) operating at 690 nm (the shortest achievable wavelength that overlapped with the absorption spectrum of ${\mathrm{AlPcS}}_{4}$) with 10 Hz repetition and 3 ns pulse full width at half maximum was used for ${\mathrm{AlPcS}}_{4}$ excitation. The laser output was coupled to a 5-mm multimode fiber bundle and provided pulses with energy of 5 mJ/pulse. A bandpass filter 690/10 nm was used to clean up the laser emission. A divergent beam from the lightguide illuminated the sample with $0.34\;\mathrm{mJ}/{\mathrm{cm}}^{2}/\text{pulse}$(average power $3.4\;\mathrm{mW}/{\mathrm{cm}}^{2}$). The TRIGGER OUT of the laser was directly connected to the TRIGGER IN of the camera for synchronization. Luminescence from the sample was collected by objective lens (Nikon #f 1/1.2, diameter 49 mm) equipped with a spacer ring to shorten the working distance to ∼15  cm. The luminescence was filtered with 725/50 bandpass and FGL9 (710-nm edge) long-pass filter to remove the excitation light and protect the intensified camera from a potential damage. After every laser pulse, the camera’s gate opens at a specific delay time and remains opened for the duration of the gate width. Several of such exposures are typically accumulated on the CCD before the frame is read out. To capture an overall DF image *in vivo*, gate delay of 1  μs and gate width of 20  μs were used. To obtain DF lifetime image and kinetics *in vivo*, sequences of 6 to 12 frames with 3  μs gate width and increasing delay were captured, with 10 on-CCD accumulations per frame before the read-out. The typical collection time was then 1 to 2 s for one frame and ∼20  s for the time-resolved sequence of images. An intensifier gain of 50× was mostly used for DF (maximum is 100×). PF is much stronger than DF and hence gain of 1× was used for PF. To measure time-resolved PF, the gate width was decreased to its minimal value (3 ns), and a sequence of 16 frames with delay increasing in 3-ns steps was recorded. Regular PF images were captured using a 48-μs gate width and 3-ns delay after the center time of the laser pulse to avoid overexposure by the scattered light. The average fluence rate during imaging was relatively small ($3.4\;\mathrm{mW}/{\mathrm{cm}}^{2}$) and imaging itself delivers only a small radiant exposure ($<1\;J/{\mathrm{cm}}^{2}$) to the sample. To deliver a PDT light dose, a continuous 635 nm laser beam (World Star Tech, compact multiwave laser) was used with power of $50\;\mathrm{mW}/{\mathrm{cm}}^{2}$ directed on the area of $0.25\;{\mathrm{cm}}^{2}$, which resembled the size of tumor, providing fluence rate of $200\;\mathrm{mW}/{\mathrm{cm}}^{2}$.

The DF kinetics from *in vivo* samples is mostly nonexponential, which makes it challenging to describe the decay by a single lifetime. In DF lifetime images, a single “effective” value of DF lifetime was estimated in each pixel by first calculating a simple intensity-weighted average of emission times in each pixel

$$\overset{˜}{\tau }(x,y)=\frac{\sum_{i}t_{i}I(t_{i},x,y)}{\sum_{i}I(t_{i},x,y)}$$ (3)

and then we found in a lookup table what would have to be the lifetime τ of a single exponential decay to yield $\overset{˜}{\tau }$ for that specific setting of gate delays and widths. Least-squares fitting with a single exponential provides similar results, but the above method was more robust and faster.

## 3 Results

### 3.1 Singlet Oxygen Feedback Delayed Fluorescence in Tissue Phantoms

**Fig. 2:**
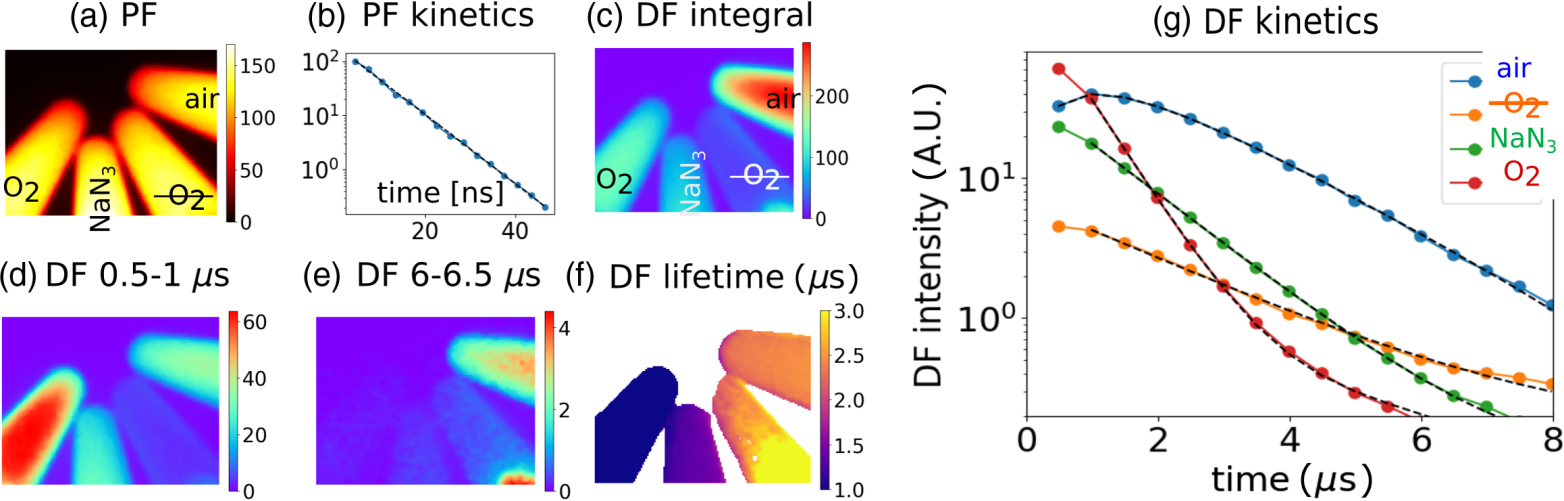
${\mathrm{AlPcS}}_{4}$ 50  μM in tissue phantoms in tubes. (a) PF image. From left to right: oxygen-saturated; with addition of ${\mathrm{NaN}}_{3}$; oxygen-depleted; and air-saturated samples. (b) PF decay kinetics, (c) overall DF emission in gate interval of 0.5 to 8  μs, (d) the first time-gated frame at 0.5 to 1.0  μs, e) the 12th time-gated frame at 6.0 to 6.5  μs, and (f) DF lifetime image calculated from the time-gated sequence of frames using Eq. (3). (g) DF kinetics fitted with two exponentials (black dashed line).

First, the validity of the method was tested on 50  μM ${\mathrm{AlPcS}}_{4}$ dissolved in tissue phantoms consisting of 1% intralipid and 1% blood in PBS. Figure 2(a) shows four tubes with the following contents (from right to left): (1) air-saturated sample alone, (2) the sample after removing oxygen by a glucose oxidase/catalase scavenging system,^46^ (3) the sample with addition of 50 mM ${\mathrm{NaN}}_{3}$, a specific quencher of O21, and (4) the sample bubbled with oxygen for 5 min. The PF intensity from all the samples has roughly the same intensity and identical lifetime of 6.4 ns. In contrast, DF from individual samples differs strongly. A DF image sequence with 0.5-μs gate width and delays increasing from 0.5 to 8  μs was captured. The DF kinetics of the air-saturated sample displays a rise–decay shape (with rise time of 0.8  μs and decay time of 1.7  μs), which is a footprint of the SOFDF mechanism.^20^^,^^25^ The DF is strongly quenched by ${\mathrm{NaN}}_{3}$, which proves that SOFDF takes place. The oxygen-saturated sample has a high initial amplitude of SOFDF signal, which however decays quickly (0.6  μs) due to triplet lifetime shortening by oxygen quenching. The oxygen-depleted sample shows poor intensity, which further supports that DF is oxygen-mediated. The lifetime is prolonged only little, to 2.2  μs, indicating that apart from oxygen quenching there is another significant deactivation pathway for triplets.

### 3.2 Fluorescence of Aluminum(III) Phthalocyanine Tetrasulfonate in a Mouse

**Fig. 3:**
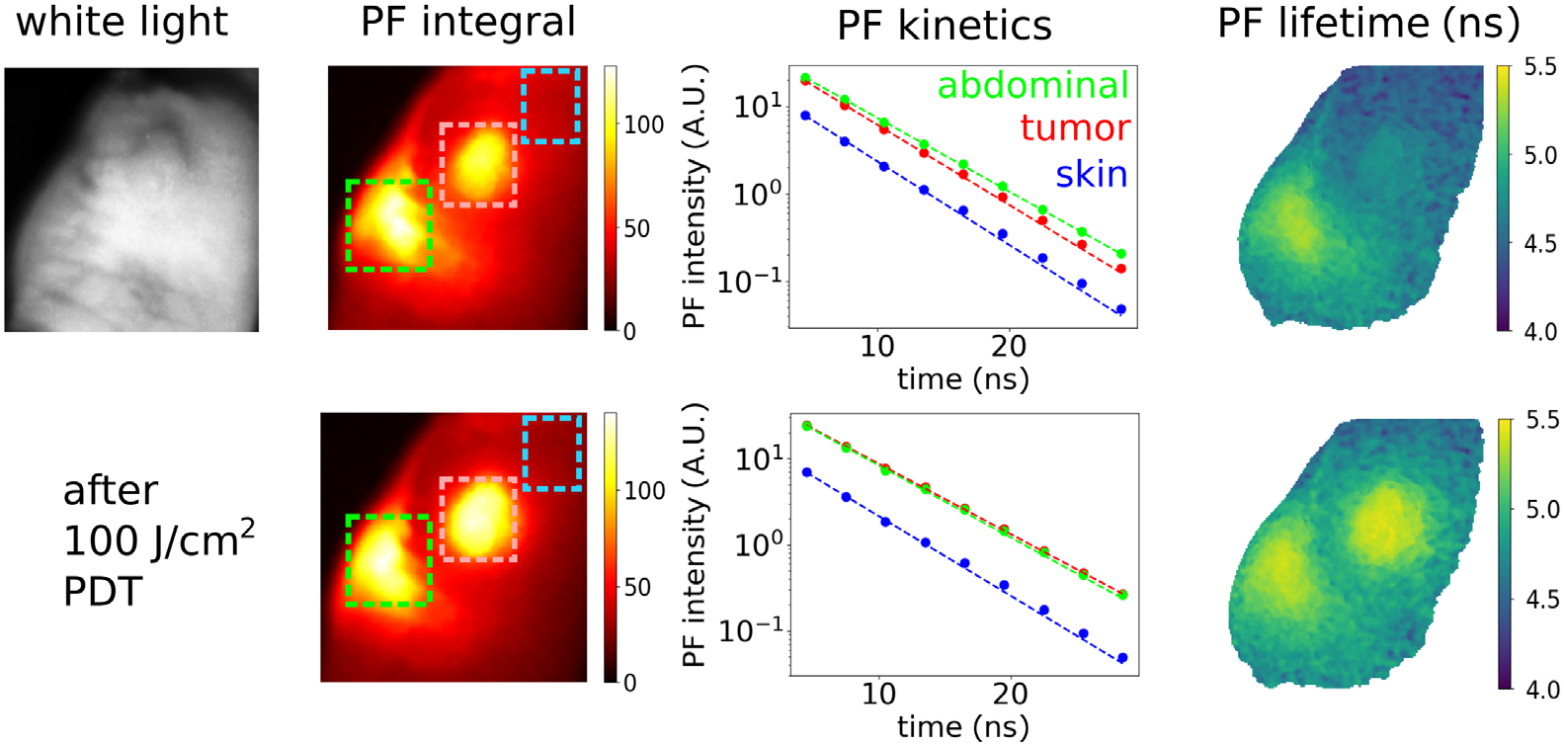
Mouse with a subcutaneous tumor after ${\mathrm{AlPcS}}_{4}$ injection. First row: before irradiation, second row: after $100\;J/{\mathrm{cm}}^{2}$ irradiation. The third column displays PF kinetics from tumor, skin, and abdominal area as marked by rectangles in the PF image.

Figure 3 displays the PF images of a tumor-bearing mouse that was intravenously injected with ${\mathrm{AlPcS}}_{4}$. This showed excellent accumulation in the tumor, but it was also present in the abdominal area and to a lesser extent in other tissues including skin, which is common after systemic administration of photosensitizers. The PF lifetime image was calculated from a series of time-gated images with 3 ns gate width and increasing delay. The lifetime of PF is clearly longer in the abdominal area (5.2 ns) than in other parts of the body, including the tumor (4.7 ns), which indicates that the microenvironment and immediate surroundings of ${\mathrm{AlPcS}}_{4}$ molecules is different, with less quenching in the abdominal area. After delivery of PDT light dose to the tumor, the PF intensity increased and PF lifetime got longer from 4.7 to 5.2 ns, thus reaching the same value as in the abdominal area. This indicates that lysosomal permeabilization in the tumor took place,^37^^,^^38^^,^^47^ releasing ${\mathrm{AlPcS}}_{4}$ from lysosomes to cytosol and changing the immediate surroundings of ${\mathrm{AlPcS}}_{4}$ molecules, thus affecting their spectral properties. Although the PF lifetime increase seems to be only subtle, it can be easily imaged due to a good signal-to-noise ratio.

By studying a group of 10 mice, it was found that the enhancement of PF amplitude by PDT treatment of the tumor was quite variable, but mostly within the range of 20% to 80%. In contrary, the PF lifetime was usually increased by 0.5 ns, from 4.7 to 5.2 ns. Radiant exposures below $3\;J/{\mathrm{cm}}^{2}$ did not induce measurable changes in PF, whereas exposures of 10 to $20\;J/{\mathrm{cm}}^{2}$ usually caused significant changes in PF lifetime and intensity. Further changes in PF after delivering more than $30\;J/{\mathrm{cm}}^{2}$ were small.

### 3.3 Singlet Oxygen Feedback Delayed Fluorescence in a Live and Dead Mouse

**Fig. 4:**
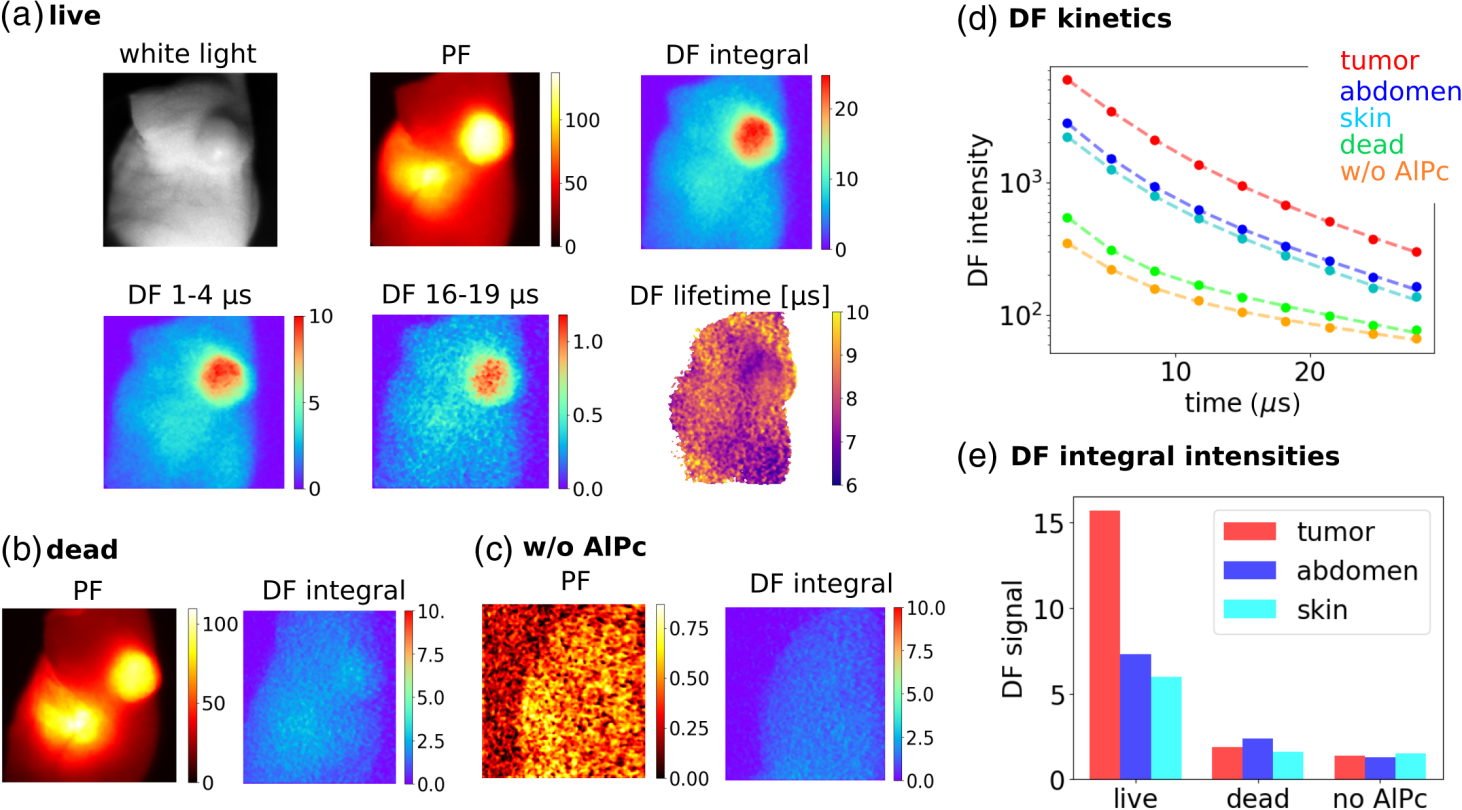
PF and DF images from a mouse with tumor. (a) Live mouse, the DF lifetime image was calculated according to Eq. (3). (b) Dead mouse 10 min after the sacrifice, (c) a control mouse without ${\mathrm{AlPcS}}_{4}$ injection, (d) DF kinetics from different parts of the body, and (e) comparison of DF signal intensities in different parts of the body for live, dead, and control mice.

Further, we examined basic properties of DF emission *in vivo*, and most importantly, we observed a dramatic loss of DF signal when the animal was sacrificed, thus supporting the hypothesis that the DF emission is indeed oxygen-mediated. These results are shown in Fig. 4. Interestingly, the DF signal from the abdominal area was comparatively much weaker than the PF signal, whereas DF from the tumor was very bright [Fig. 4(a)]. Therefore, PF and DF images provide different information. A probable explanation is that SOFDF, as a second-order process, is sensitive to the “local” concentration rather than the “average” concentration, and local microenvironments in tumor and abdominal area are different, as indicated by the different PF lifetimes (Fig. 3). DF time-resolved sequences with a gate width of 3  μs and delay increasing from 1 to 16  μs were captured, and Fig. 4(a) displays the first and fifth frames for illustration. Based on this sequence, DF lifetime image was calculated, providing DF lifetime of ∼8  μs in the tumor and slightly longer lifetimes (∼9  μs) in some other tissues. The measured DF lifetimes differed slightly in different animals (n=5) within the range of 8 to 11  μs.

Figure 4(b) displays the PF and DF emissions from the same mouse 10 min after it was sacrificed, which caused rapid depletion of intracellular oxygen in tissues. While the PF changed only little, the DF emission from the tumor dropped dramatically, more than 6×. This experiment clearly proves that the DF emission is produced by the singlet oxygen feedback mechanism and not by other DF mechanisms, which on the contrary are enhanced in the absence of oxygen.^25^ The experiment with a live/dead mouse was done with three different animals and provided consistent results.

Finally, Fig. 4(c) displays the signal from a live mouse that was not administered ${\mathrm{AlPcS}}_{4}$. This shows that the majority of the weak signal from the dead animal (previously injected with ${\mathrm{AlPcS}}_{4}$) was probably due to autofluorescence of the tissue and not related to ${\mathrm{AlPcS}}_{4}$ emission. Lifetimes of these residual DF signals were 13 to 17  μs.

### 3.4 Photodynamic Therapy Effect and Visualization of Lysosome Permeabilization

Subsequently, the effect of PDT and lysosome permeabilization on the parameters of DF was investigated. Tumors of mice were exposed to increasing light doses delivered by a 635-nm laser at 40 or $200\;\mathrm{mW}/{\mathrm{cm}}^{2}$ (with a smaller diameter beam).

**Fig. 5:**
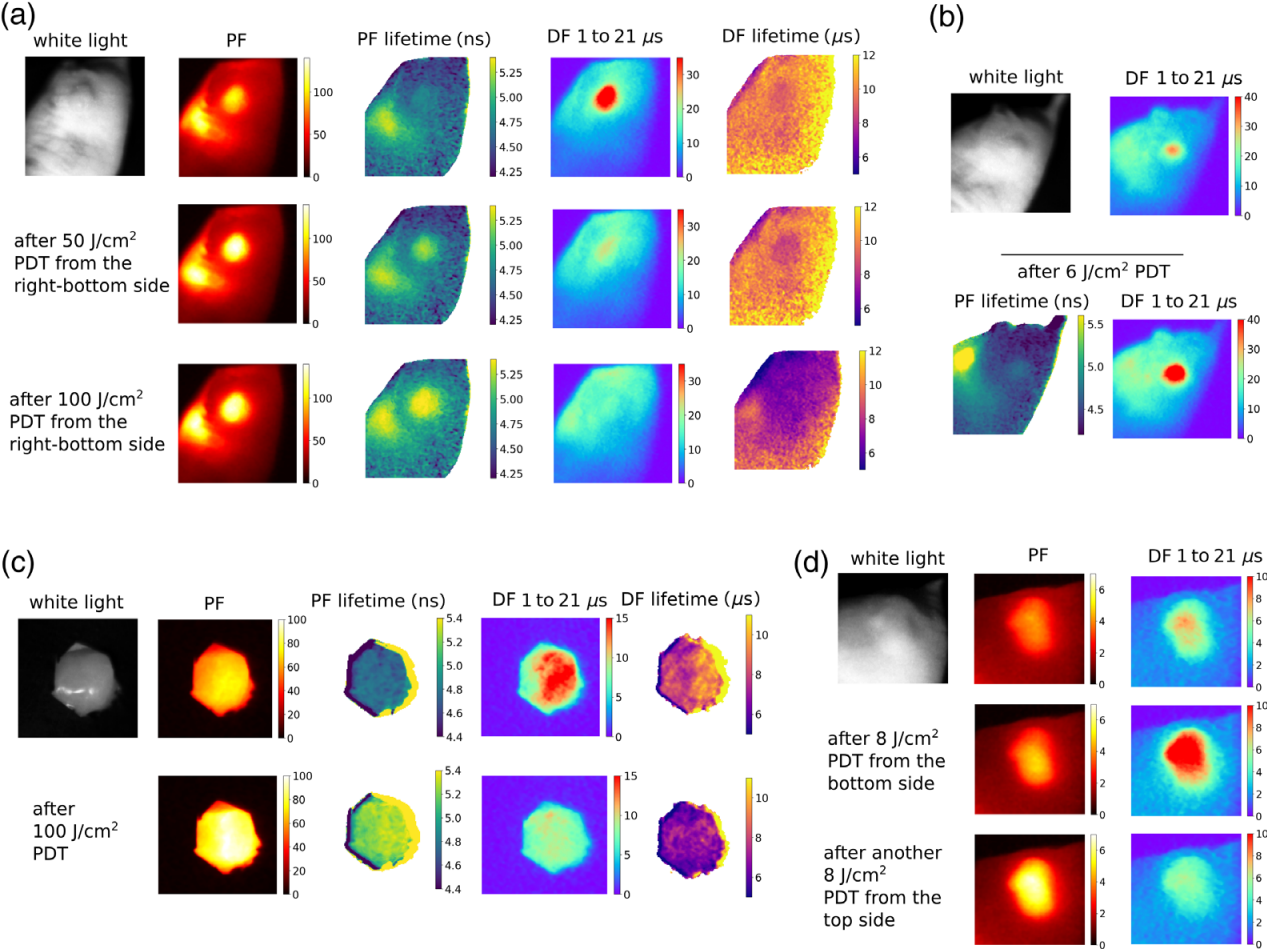
PF and DF intensity and lifetime images from a mouse during PDT irradiation of the tumor with different radiant exposures. (a) The effect of 50 and $100\;J/{\mathrm{cm}}^{2}$ on DF and PF intensities and lifetimes. (b) Small radiant exposure of $6\;J/{\mathrm{cm}}^{2}$ caused an enhancement of DF while PF lifetime did not show marks of lysosome permeabilization. (c) Tumor was exposed by removing the skin, and the surroundings of the tumor were covered with a black tape to prevent any potential light scattering from outside of the tumor. (d) Irradiation of the tumor from the bottom side with $8\;J/{\mathrm{cm}}^{2}$ and then from the top side.

In contrary to PF, larger light doses and lysosome permeabilization led to fading of DF [Fig. 5(a)]. After delivery of a larger light dose, the loss of DF proceeds also in dark without further irradiation (data not shown). The DF from the tumor usually faded out and gradually converged to the intensity of the signal from the surroundings, whereas the DF from the skin and surrounding tissues often increased a little, which could be due to the small light dose of scattered light that it receives, as shown later. Even though the DF signal drops and the tumor contrast decreases, there is still plenty of DF emission (high above the background levels), indicating that the generated O21 can still be detected. The PF emission from the tumor does not fade out even after prolonged periods of irradiation due to the excellent photostability of ${\mathrm{AlPcS}}_{4}$. The drop of the SOFDF signal after the lysosomal release could be explained by the fact that SOFDF is a second-order process in the concentration of triplet states, and as such it is weakened after dilution into cytoplasm. In addition, it is interesting to note that the DF lifetime is slightly shortened during the PDT, which is unexpected, because lengthening of lifetime due to oxygen depletion is rather anticipated.^21^

Figure 5(b) shows that a relatively small radiant exposure ($6\;J/{\mathrm{cm}}^{2}$), can lead, on the contrary, to a remarkable increase in DF signal (by 30% to 100% in different animals, n=3). In this specific case, this exposure did not induce an appreciable lysosome permeabilization in the tumor, as can be seen from the absence of a lifetime increase in the PF lifetime image.

It was verified that the time-resolved DF and PF signals could be imaged also from an exposed tumor after removal of the skin, as shown in Fig. 5(c), giving results consistent with skin-overlaid tumors. The surroundings of the tumor were covered with black material to prevent any potential scattering from tissue outside of the tumor.

Figure 5(d) shows PF and DF from a tumor that was irradiated first from the bottom side and then from the top side, thus simulating an inhomogeneous illumination, which is a common problem in PDT due to scattering and varying depth of the tumor. This illustrates the power of imaging over the point detection because different parts of the tumor are in different stages of PDT, as can be seen in the PF and DF images.

The experiments shown in Figs. 5(a)–5(d) were performed with groups of mice counting five, three, three, and three animals, respectively, and provided qualitatively consistent results. The experiment in Figs. 5(d) was done with different camera settings and therefore the absolute values of signal intensity shown in the images are not directly comparable to the images in remaining panels. Measurements of PF and DF intensities in a group of five mice showed that luminescence intensities from tumors in different individuals varied up to a factor of 2. Nevertheless, the qualitative observations described above were consistent across different animals.

### 3.5 Singlet Oxygen Image

An image of SOFDF from a mouse with tumor was presented in Fig. 4(a). It is interesting to investigate what is the relation of such SOFDF image to the O21 image. SOFDF emission comes from the encounter of the excited triplet state with O21 and therefore the SOFDF emission rate is proportional to the product of the O21 concentration and triplet state concentration, as shown in Eq. (1). This implies that

[O21](t)∝ISOFDF(t)/[T1](t).

**Fig. 6:**
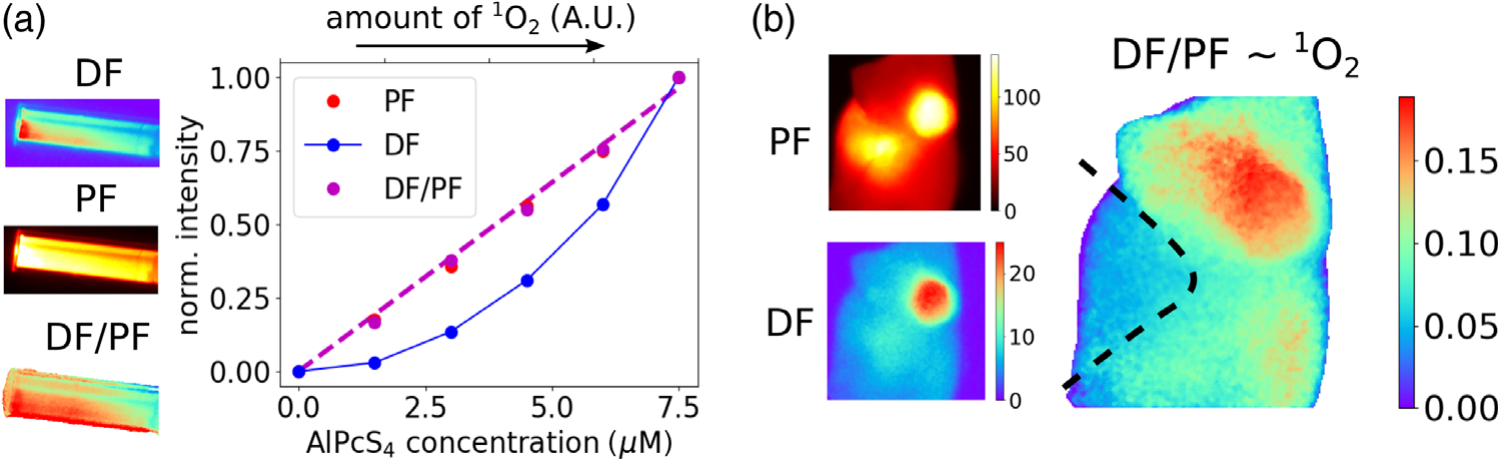
(a) ${\mathrm{AlPcS}}_{4}$ in PBS in cuvettes at concentrations ranging from 0 to 7.5  μM. The graph shows the dependence of the average PF, DF, and DF/PF signal on the ${\mathrm{AlPcS}}_{4}$ concentration and amount of the generated O21. (b) O21 image approximated as the ratio of DF and PF. The plotted values of DF/PF ratio do not correspond to the absolute ratio of the emission rates, because the DF and PF images were captured with different camera settings (gain etc.). The dashed line indicates that the SOFDF intensity from the abdominal area cannot be easily compared to the SOFDF intensity in the rest of the body due to the very different microenvironment.

The initial concentration of triplets generated after a laser pulse is usually proportional to the PF intensity, because we assume that the intersystem crossing rate and the radiative fluorescence rate are in a fixed proportion. Then the O21 image could be approximated by taking the SOFDF image and dividing it pixel by pixel with the PF image. This approach is validated in Fig. 6(a). A series of solutions of ${\mathrm{AlPcS}}_{4}$ in PBS in cuvettes was prepared, with concentrations gradually increasing from 0 to 7.5  μM. Absorbances of all these samples were below 0.1 to avoid an inner filter effect and other artifacts. Under such conditions, the amount of generated triplet states scales linearly with the ${\mathrm{AlPcS}}_{4}$ concentration, and so does the amount of generated O21. (This is because the amount of O23 is much larger than the amount of triplet excited states, and a vast majority of triplets is quenched by oxygen to give rise to O21, thus making the amount of the generated O21 proportional and very similar to the amount of triplet states.) We can see in the graph in Fig. 6(a) that the ratio of intensities $I_{\mathrm{SOFDF}}/I_{\mathrm{PF}}$ is indeed linearly proportional to the amount of the generated O21.

Figure 6(b) then shows the O21-based image of a mouse reconstructed by dividing the DF image with a PF image pixel by pixel [background level measured in Fig. 4(c) was subtracted from the DF image first]. However, caution must be exerted when interpreting such an image. As shown earlier, the PF lifetime was different in the abdominal area, which indicated a different local microenvironment in the surroundings of ${\mathrm{AlPcS}}_{4}$ and likely also a different local concentration of excited states. Therefore, the SOFDF from the abdominal area was not comparable to SOFDF from the tumor and other parts of the body. This is indicated by the dashed line in Fig. 6(b), which approximately delimits the abdominal area.

## 4 Discussion

### 4.1 Prompt Fluorescence Lifetimes

The following values of PF lifetimes *in vivo* were found: 5.2 ns in abdominal area, 4.7 ns in other parts of the body before irradiation, and 5.2 ns after lysosome permeabilization. PF lifetime of 6.2 ns was measured in a PBS solution at pH 7 (data not shown). Although there may be a systematic bias up to ∼0.5  ns due to the relatively broad laser pulse (3 ns) and gate width (3 ns), these values are in line with the previously reported PF lifetimes in solutions (5 to 6 ns) and cells (4 to 5 ns).^42^^,^^43^^,^^48^ PF lifetime in acidic pH 4.1 was reported to be shorter by 0.5 ns, when compared to neutral pH 7.^48^ This is one of the possible reasons for the PF lifetime increase after lysosomal permeabilization, because the interior of lysosomes is known to be more acidic than cytosol. It was also reported that aggregates of ${\mathrm{AlPcS}}_{4}$, which may be favored at high local concentrations inside lysosomes, have a short PF lifetime (<1  ns),^42^ but the time resolution of our time-gating (3 ns) does not allow to evaluate reliably such a fast luminescence component. While the increase of PF intensity certainly reports on lysosome permeabilization, it has a rather large variability, and time-gated lifetime imaging could be quite a robust technique to complement intensity measurements.

### 4.2 Intensity of Singlet Oxygen Feedback Delayed Fluorescence Signal

The SOFDF-based O21 image in Fig. 6(b) was obtained with capture time of 6 s at 10 Hz laser repetition rate, and total radiant exposure of $20\;\mathrm{mJ}/{\mathrm{cm}}^{2}$, which is 3 orders of magnitude below the therapeutic radiant exposures. This illustrates the feasibility of real-time imaging during PDT. Moreover, the capture time and radiant exposure could be further significantly decreased by shifting the excitation from 690 nm to a shorter wavelength (630 to 660 nm). This would increase the absorption and would enable us to capture the main fluorescence peak around 680 nm, thus increasing the DF signal at least by a factor of 3 [Fig. 1(d)]. Excitation at 690 nm was used here because it is the minimal achievable wavelength with our tunable laser that overlaps with ${\mathrm{AlPcS}}_{4}$ absorption spectrum.

By comparing the intensity of PF and DF after the laser pulse, it was found that the integral DF from the tumor was 2000 to 6000× weaker than the integral PF. With PF quantum yield of ∼0.4,^42^^,^^43^ we get a DF quantum yield of approximately $10^{-4}$ in the conditions of our experiment (it has to be remembered that the quantum yield of SOFDF, a second-order process, is a function of excitation intensity and photosensitizer concentration). Although this may seem a small number, the signal can be readily detected by an intensified gated camera, and it is about 3 orders of magnitude larger than the quantum yield of O21 luminescence.^5^ Moreover, the detection in the visible spectral region is technically less demanding than in the near-infrared.

### 4.3 Comparison with Results Obtained in Cell Cultures In Vitro

When comparing our earlier-reported results in monolayer of cells *in vitro*^22^ with those obtained here in mouse *in vivo*, several similarities and several differences can be noticed. In both cases, lysosome permeabilization after irradiation usually caused an increase in PF intensity and a decrease in DF intensity. However, the decrease in DF was much more dramatic *in vitro*, whereas *in vivo* the DF signal is still high above the background levels even after prolonged irradiation. Moreover, a substantial initial increase in DF intensity was observed *in vivo* after a small radiant exposure, whereas this effect was much weaker or nonexistent *in vitro*. The ${\mathrm{AlPcS}}_{4}$ DF decay times *in vitro* were found to grow slightly after lysosome permeabilization (from 3.7 to 4.1  μs), and more so when using ionic porphyrins.^21^ In contrary, *in vivo*, the DF of ${\mathrm{AlPcS}}_{4}$ decayed with lifetimes 7 to 11  μs and the lifetime slightly decreased during irradiation. This is rather unexpected because triplet lifetime is supposed to increase due to oxygen depletion during irradiation. In solutions, SOFDF lifetimes were found to report on oxygen concentration in the sample.^20^^,^^26^ It would be very valuable to be able to infer such information also *in vivo*, but unfortunately the interpretation of the SOFDF decay times in this work is unclear. It is possible that in this specific case, the quenching by oxygen is not the most important triplet decay pathway.

### 4.4 Interpretation of Singlet Oxygen Feedback Delayed Fluorescence Signal: Benefits and Limitations

We showed that the detected delayed emission can be indeed attributed to SOFDF. In tissue phantoms, this was shown by (1) quenching of DF by ${\mathrm{NaN}}_{3}$, (2) absence of DF in absence of oxygen, (3) enhancement of DF amplitude at higher oxygen concentration, and (4) rise–decay shape of the kinetics. *In vivo* in mouse, this was confirmed by a dramatic loss of DF signal after oxygen depletion achieved by sacrificing the animal (other types of DF are known to be enhanced at lower oxygen instead). The short rise-time of the SOFDF *in vivo* did not allow us to resolve the rise of the SOFDF kinetics.

In Fig. 2, we can notice that the overall DF emission intensity in tissue phantoms is stronger for the air-saturated sample than for the oxygen-saturated one, even though in the latter case the total amount of the generated O21 must be equal or somewhat larger. This illustrates an important caveat of the SOFDF-based sensing of O21: when the lifetime of O21 gets comparable or even larger than the triplet lifetime, which happens typically at high concentration of O23, the O21 population can “outlive” the triplet state population, and finding a collision partner for SOFDF becomes improbable. This typically leads to lowering of the integral SOFDF emission at high O23 concentrations, as discussed elsewhere.^25^ However, this scenario is not common *in vivo*, where the oxygen concentration is significantly lower and hence the triplet lifetime is longer than in air-saturated solutions (many microseconds),^49^ and O21 lifetime is shorter, below 1  μs.^29^ Under such conditions, SOFDF integral intensity is an increasing function of the amount of the generated O21.

Another caveat was illustrated in Fig. 6(b). It was suggested that the O21 image can be best approximated by dividing the SOFDF image with the PF image to account for different average concentrations of the photosensitizer in different regions. However, it was noted that the intensity of the SOFDF/PF image still cannot be easily compared between two regions with a very different local surroundings of the ${\mathrm{AlPcS}}_{4}$ molecules. To explain this more clearly, let us assume that the average concentrations of triplets and also the amount of generated O21 in two regions of the body are the same, but in one region the triplets are localized in lysosomes at very high local concentrations, whereas in the other region triplets are spread in the cytoplasm at a much smaller local concentration. The SOFDF signal will be much larger in the first region, because the probability of interaction between O21 and the triplet is higher, whereas in the second region the O21 is much more likely to be deactivated by concurrent chemical and physical quenching instead. As a result, SOFDF signals differ, even though the amounts of triplets and O21 were the same. For the same reason, the SOFDF signal drop after lysosome permeabilization does not necessarily mean that a smaller amount of O21 is produced, because the local concentration of triplets is dramatically changed and thus the comparison of SOFDF before and after the permeabilization is troublesome. Therefore, it must be always remembered that SOFDF rate is proportional to the product of the triplet and O21 local concentrations and not average concentrations. As such, SOFDF reports only on the local concentration of O21 in the immediate vicinity of the photosensitizer molecule and not on the average concentration of O21 over the whole cell.

This is certainly an important shortcoming, but it must be noted that a very similar problem will be encountered with any indirect method relying on a fluorescence probe. The amount of signal from the fluorescence probe will be proportional to the number of interactions between O21 and the probe molecules, and therefore dependent on the inherently heterogeneous local microscopic concentration of the probe, which cannot be easily determined nor controlled. Moreover, the distribution of the probe in the individual cellular compartments will be different from the distribution of the photosensitizer, leading to very different probe responses depending on where in the cell the O21 is produced (the intracellular diffusion radius of O21 is only ∼ 100  nm^29^ and hence it has to be sensed locally in the site of its production). With SOFDF, the colocalization of the probe with the site of O21 production is not an issue. To conclude, the dependence of the signal response on the local microenvironment is always present in any indirect method, and SOFDF is not an exception. Only the direct detection of the O21 near-infrared phosphorescence emission will not suffer from these issues. However, it is interesting to note here that, even in the case of the steady-state direct detection, one has to be cautious, because the amount of phosphorescence increases with the lifetime and the radiative rate of O21, which both depend on the local microenvironment.^29^ To account for this at least partly, time-resolved detection would have to be used. Although this can be achieved with a point detection,^6^ time-resolved imaging would be quite challenging, given the extremely low signal levels.

Comparison of benefits and limitations of SOFDF-based O21 detection and other methods has been discussed in detail in our earlier works.^21^^,^^25^ Despite the caveats discussed here, we believe that SOFDF-based sensing of O21 could be a valuable tool, given that all other techniques suffer from serious problems. To our knowledge, O21-based images of tumors with a comparable radiant exposure, capture time, and quality have never been reported before. Although SOFDF cannot be applied to all photosensitizers, it was reported that a wide range of other photosensitizers are also capable of SOFDF emission (${\mathrm{TPPS}}_{4}$, TMPyP, porphycenes, eosin, rose Bengal, etc.),^20^^,^^26^ including protoporphyrin IX,^27^ which is currently the most frequently used photosensitizer in clinical PDT (it is formed endogenously in cells after administration of δ-aminolevulinic acid). Therefore, SOFDF-based singlet oxygen sensing could be potentially performed with a wide range of photosensitizers.

## 5 Conclusions

It was demonstrated that imaging of SOFDF is feasible *in vivo* in tumors of mice injected with 2.5 mg/kg ${\mathrm{AlPcS}}_{4}$, using a 690-nm pulsed excitation with $0.34\;\mathrm{mJ}/{\mathrm{cm}}^{2}/\text{pulse}$ and an intensified microsecond-time-gated camera for detection. It was verified that the emission is indeed O21-mediated. The quantum yield of SOFDF emission *in vivo* was found to be in the order of $10^{-4}$ in our experimental conditions, which is 3 orders of magnitude larger than the quantum yield of the direct O21 phosphorescence, and as such SOFDF imaging could be a valuable technique for monitoring of O21 *in vivo*. SOFDF is exhibited by a wide range of different photosensitizers, thus making this approach of a broader interest, although not applicable in every situation. It was also illustrated that comparison of SOFDF intensities from two different types of tissue with substantially different microenvironment is not straightforward and caution has to be exerted. The time-gated approach used here enabled us to capture also lifetime images of PF with nanosecond time resolution, and it was shown that PDT-induced lysosome permeabilization can be nicely visualized by the change in fluorescence lifetime.
